# Localized Hydrogel-Based Therapeutics: A Strategic Approach to Mitigating Postoperative Recurrence in Breast Cancer

**DOI:** 10.3390/polym18172097

**Published:** 2026-08-29

**Authors:** Kiran Kainat, Muhammad Saif Ur Rahman, Muhammad Shoaib, Shanshan Xu

**Affiliations:** 1 Institute for Advanced Study, Shenzhen University, Shenzhen 518060, China; 2551063001@mails.szu.edu.cn (K.K.); fiasanar@temedical.com (M.S.U.R.); 2 Sustainable Materials Research Center (SUSMAT-RC), College of Chemical Sciences and Engineering (CCSE), Mohammed VI Polytechnic University (UM6P), Benguerir 43150, Morocco; muhammad.shoaib@um6p.ma

**Keywords:** cancer, breast cancer, hydrogels, local drug delivery, postoperative recurrence, biomaterials, immunotherapy

## Abstract

Cancer remains one of the leading causes of death globally, and breast cancer is the most frequently diagnosed cancer in women. Even though surgical excision is the main treatment of solid tumors, recurrence and metastasis to another location postoperatively provide lasting therapeutic challenges. Systemic adverse effects and a lack of sufficient drug bioavailability at the resection site can often hinder traditional adjuvant modalities, especially chemotherapy and radiotherapy. To address these drawbacks, localized drug delivery platforms have gained a lot of interest, and hydrogels have emerged as highly beneficial systems. Hydrogels are three-dimensional hydrophilic polymer networks that can be designed to be injectable and in situ-forming. They can be engineered to fit irregular surgical cavities and deliver therapeutics in a long-lasting, controlled manner. The current literature review outlines the clinical issue of tumor recurrence following surgery with a special focus on breast cancer and evaluates hydrogel-based methods of site-specific therapy. We outline the categorization, assembly, and functionalization of hydrogel systems based on natural, synthetic, and hybrid polymers, and reflect on advanced stimuli-responsive and cell-targeted formulations in the context of cancer immunotherapy. This paper presents the translational applications of hydrogel platforms to enhance postoperative management and prevent tumor recurrence by combining new advances.

## 1. Introduction

Cancer remains one of the major health issues in the world, posing a considerable percentage of health burden and mortality. Globally, it is estimated that 14.1 million new cases of cancer, as well as 8.2 million deaths, were attributed to cancer in 2012. Projection also shows that the number of people getting the disease is on a steady increase, and by the year 2025, the number of new cancer cases is projected to be almost 19.3 million every year [1,2,3].

The treatment of cancer depends on the type of tumor and the stage at which the disease is diagnosed. Recent clinical management approaches comprise surgery, radiotherapy, chemotherapy, hormone-based therapy, immunotherapy, and gene-targeted therapy [4,5]. Of them, chemotherapy is one of the most used modalities to control the progression of tumors and prevent recurrence. The basis of chemotherapeutic treatment is the use of cytotoxic agents that destroy rapidly dividing cancerous cells, but one of the biggest shortcomings of this treatment is that it lacks selectivity; hence, causing collateral damage to healthy tissues, as well as a high level of systemic toxicity [6,7].

Conversely, localized drug delivery approaches have been receiving growing interest due to their ability to deliver the therapeutic agent to the target area, thereby limiting systemic exposure and side effects. The methods also enable sustained, controlled delivery of anticancer drugs, thereby increasing therapeutic efficacy and improving treatment outcomes [6,8].

The polymers in hydrogels that are employed in breast cancer applications can be classified broadly as Natural Polymers, Synthetic and Hybrid Polymers. Their chemical structure and functional groups strongly influence hydrogel performance. Natural polymers such as collagen, gelatin, hyaluronic acid, chitosan, and alginate have good biocompatibility and biological interactions but may have relatively poor mechanical stability and batch variability. On the other hand, a synthetic polymer, such as polyethylene glycol (PEG) and poly(vinyl alcohol) (PVA), having good control over the molecular structure, mechanical properties, and degradation, can be adopted. They need to be functionalized for better biological interactions. The combination of natural and synthetic polymers that hybrid hydrogels offer can therefore give rise to optimal biological activity and mechanical stability.

The crosslinking strategies of polymeric systems further decide their properties. The formation of a 3D network using polymer chains and covalent bond interactions is referred to as chemical cross-linking, while the use of ionic interactions, hydrogen bonding, hydrophobic interactions, and polymer chains entangling each other is physical cross-linking. The network density, swelling capacity, mechanical strength, rate of degradation, and the drug-release profile of a polymer depend on its chemistry and method of crosslinking. Higher crosslinking density improves stability but reduces swelling and drug diffusion, whereas lower density promotes swelling and faster release. For postoperative breast-cancer hydrogels, indicative targets include a modulus of 1–10 kPa, swelling of 10–50 g/g, pore sizes of 50–200 μm, gelation within minutes, and persistence for 3–5 weeks with release lasting up to ~80 days. These values should be optimized for the formulation and treatment period [9,10].

Especially relevant is this relationship in postoperative breast cancer therapy, where hydrogels may be introduced into the surgical cavity following tumor excision to provide localized and sustained delivery of chemotherapeutic or other therapeutic agents. In this environment, biocompatibility, degradation behavior, mechanical properties, swelling, and release kinetics should all be considered together, as too much stability can delay degradation, and not enough mechanical integrity can hamper retention at the surgical site. As a result, proper selection of the type of polymer and the crosslinking technique can allow a drug-loaded hydrogel to remain in the postoperative cavity for a longer time, enhance local drug release, target micrometastatic residual tumor cells, and reduce systemic drug exposure.

Hydrogels, nano- and microparticles, and micellar and liposomal carriers have been widely studied for localized and efficient cancer therapy [11,12]. Hydrogels have become one of the most versatile of these as they are biocompatible, tunable, and can form in situ at the surgical site. This review gives an in-depth account of the hydrogel-based local drug delivery systems, particularly the use of the system in the prevention of postoperative recurrence, such as breast cancer. Although foundational studies on hydrogel chemistry, crosslinking, and drug delivery have been conducted across different cancer models, this review prioritizes their relevance to postoperative breast cancer [13]. General hydrogel studies are discussed only when their material properties or delivery mechanisms provide principles applicable to breast cancer resection cavities and local recurrence prevention. Breast cancer-specific studies are therefore emphasized when evaluating therapeutic performance and translational potential. Despite foundational studies of hydrogel chemistry, crosslinking, and drug delivery in cancer research, we will focus on those relevant to postoperative breast cancer in this review. General studies on hydrogels are not discussed unless the delivery mechanism or material properties elucidate principles that could be applicable to breast cancer resection cavity and local recurrence prevention. Breast cancer-specific studies are thus underscored while evaluating therapeutic efficacy and translational potential.

## 2. The Clinical Challenge: Postoperative Tumor Recurrence

### 2.1. The Problem of Residual Tumor Cells: Surgery

**Figure 1:**
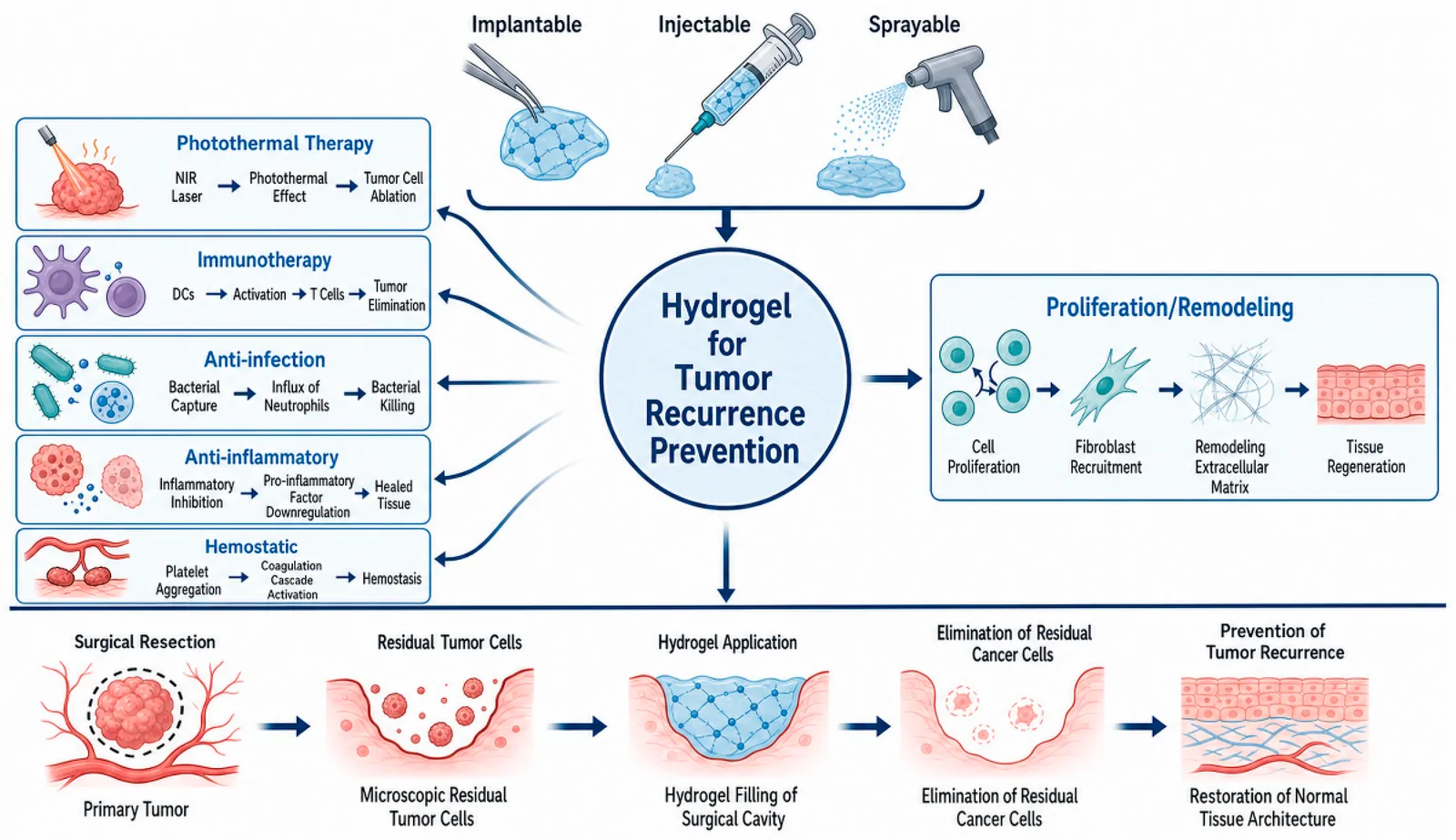
Schematic illustration shows the formulations and functionalization of hydrogels for the prevention of postoperative tumor recurrence.

In hydrogel-based tumor recurrence prevention, surgeons remove the primary tumor. Residual microscopic tumor cells may remain within the surgical cavity after resection. The hydrogel is then applied to fill the surgical cavity using suitable delivery methods. Finally, restoration of normal tissue architecture helps reduce the risk of tumor recurrence. Among the key factors that lead to recurrence is the presence of malignant cells left in adjacent tissues or at the periphery of the surgical site after tumor removal. These cells are usually very minute and may not be easily noticed during surgery. During surgery, the remaining cancer cells can enter the bloodstream, leading to local recurrence or metastasis to other organs [14]. From a biomaterials perspective, the regular resection cavity with residual tumor cells requires polymeric hydrogel systems with an ability to adapt to the site of surgery along with a sustained release of therapeutic activity. The stability, drug retention, and controlled release of a hydrogel at the resection site are dictated by polymer composition, network architecture, and crosslinking density [9] (Figure 1).

The immune system can be easily circumvented with immunosuppressants to stimulate wound healing after surgery. Reduced natural killer (NK) cytotoxicity and compromised macrophage activity are associated with increased tumor growth after surgery. Also, circulating dendritic cells (DC), which play a vital role in immunological surveillance following tumor excision, can be reduced by surgery [15].

### 2.2. The Paradox of Surgery

**Figure 2:**
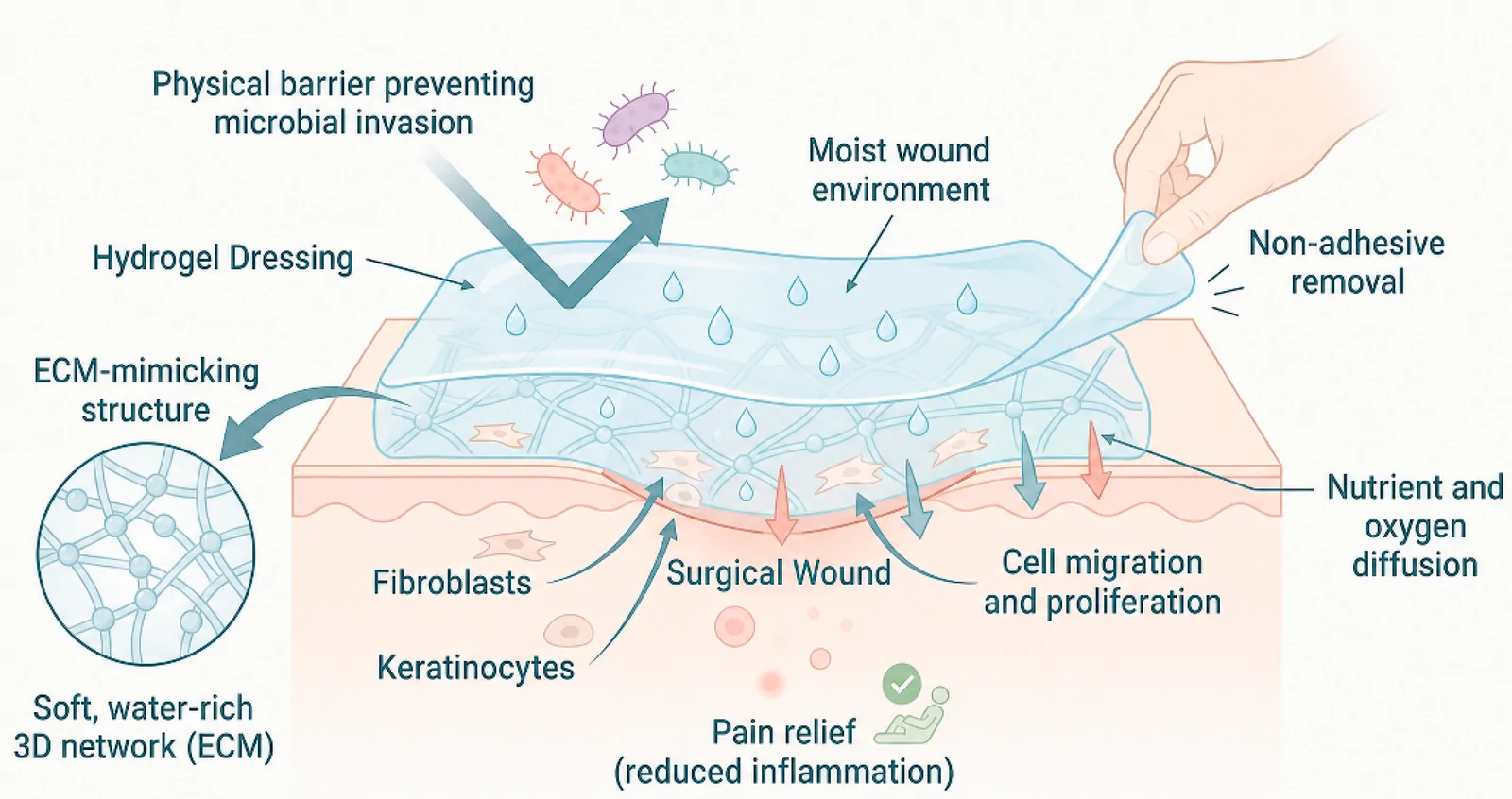
Hydrogel role in postoperative cancer wound management.

Moreover, there is growing evidence that surgical intervention per se can lead to the promotion of tumors. Surgical stress may lead to the release of catecholamines, which stimulate 2-adrenergic signaling and upregulate growth-promoting and angiogenic factors, including vascular endothelial growth factor (VEGF) [16]. Long-term increases in these mediators can drive the conversion of dormant, non-angiogenic tumor cells into actively growing, angiogenic subpopulations, leading to tumor regrowth and metastasis [17]. The importance of designing polymeric hydrogels for sustained localized delivery of therapeutic agents is further underscored by these changes. Stimuli-responsive behavior achieved through composite materials and architecture designs can be geared to adjust within the postoperative microenvironment through local physicochemical conditions to tailor drug release [10,18]. Hydrogels are intricate structures composed of hydrophilic polymer strands that rapidly swell upon exposure to water, forming a partially solid material [19]. Water constitutes over 90 percent of the hydrogel structure; therefore, a moist environment can be maintained at the wound periphery, promoting tissue healing [20]. Hydrogels are ideal for use as wound dressings due to several of their attributes (Figure 2). These are mechanical protection, form flexibility, and strong adhesion, which provide satisfactory coverage and wound protection [21]. The advantage of hydrogel-based dressings is that they can be easily tuned to incorporate growth hormones, cells, biomolecules, and antimicrobial and antibacterial agents [22]. Hydrogels have the potential to serve as temporary templates for the re-epithelialization and remodeling of chronic wounds, owing to their biodegradability and biocompatibility. Hydrogels also exhibit sufficient bioadhesion, which is critical for stability. This property maintains optimum moisture levels in the wound and enhances hemostasis. Also, hydrogels provide a versatile platform for incorporating other components, such as drugs, antibacterial and antimicrobial compounds, and other biomolecules, thereby enhancing their overall wound-healing-promoting properties [23].

### 2.3. Limitations of Adjuvant Systemic Therapies

To prevent postoperative recurrence, adjuvant therapeutic methods such as chemotherapy, immunotherapy, and targeted therapy are usually used after tumor resection to destroy the remaining malignant cells. Nevertheless, such traditional treatments usually target the whole body, which means that there will be no specificity in their distribution, poor drug bioactivity, and a significant level of toxicity to normal tissues. These limitations significantly limit their therapeutic effectiveness Table 1. As a result, there is an urgent need to develop more efficient and targeted postoperative treatment programs that will reduce recurrence rates and minimize adverse effects on the system [24,25]. Some possible adverse effects of surgical treatments include infections, deterioration in quality of life, and inability to recover after surgery. Multiple current and past studies indicate that there is no survival advantage of chemotherapy over anticipated clinical care [26,27]. The risk of complications after surgery can be increased by certain illnesses, including heart disease, blood disorders, obesity, asthma, other lung ailments, chronic obstructive pulmonary disease (COPD), and renal issues [28]. The development of polymer-based local delivery platforms can help to address these limitations, wherein polymer chemistry and crosslinking can be tuned to enhance tissue retention, drug loading, biodegradation, and release kinetics. The hydrogel must maintain localization to the resection cavity in postoperative breast cancer and allow sustained therapeutic exposure due to such material properties [29,30].

## 3. Biomaterials: An Introduction

### Biomaterials in Oncology

According to Biomaterials, artificial or naturally produced materials that are manufactured to interact with biological systems to treat, identify, or prevent disease [34]. Metals, ceramics, polymers, and composites are listed among them and are broadly used in biomedical applications due to their tunable physicochemical properties [35,36]. In the field of oncology, the use of biomaterials for local and controlled delivery of drugs has received more attention, especially for postoperative cancer treatments [37,38]. Through our research in the lab, we aimed to develop a simulated tumor environment that can be utilized in the laboratory. Hydrogels are three-dimensional networks made of natural or synthetic polymers. There is high water content in hydrogels. They resemble the extracellular matrix in chemical composition and structure and interact with surrounding cells [39]. Hydrogel preparations can also be used as dressings to maintain a moist environment, protect against microbial invasion, and prevent tissue damage on removal [40,41,42]. These agents can effectively be used for postoperative therapeutic, site-specific cancer treatment by encapsulation and local release [43].

## 4. Hydrogels as a Platform for Localized Drug Delivery

### 4.1. Importance of Hydrogel Drug Delivery

**Figure 3:**
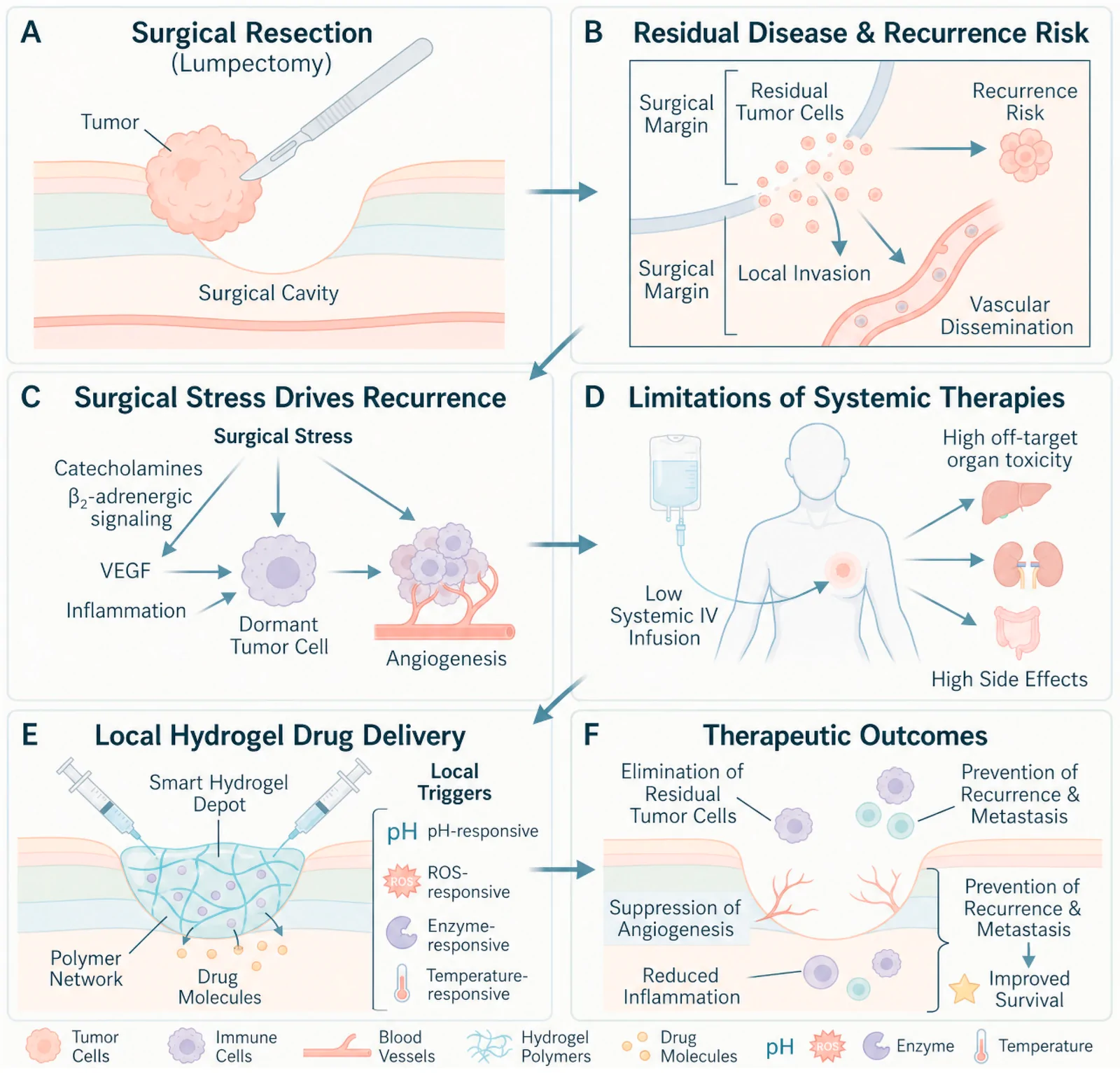
Tumor recurrence and metastasis following surgical resection. (**A**) Surgical resection. (**B**) Residual tumor cells and recurrence risk. (**C**) Surgical stress and angiogenesis. (**D**) Limitations of systemic therapy. (**E**) Local hydrogel-based drug delivery. (**F**) Therapeutic outcomes of hydrogel treatment.

Figure 3 summarizes the role of local hydrogel therapy after tumor resection. Surgical resection (A) removes the primary tumor but may leave residual cells (B), while surgical stress can promote recurrence through inflammation and angiogenesis (C). Systemic therapies may cause off-target toxicity and side effects (D). Local hydrogel delivery (E) enables controlled, stimulus-responsive drug release at the surgical site, helping eliminate residual tumor cells and suppress recurrence, angiogenesis, and inflammation (F) [44]. This localization system improves drug accumulation at the target site, enables prolonged, regulated release in the complex extracellular milieu, and greatly reduces off-target toxicity [45]. In addition, the high drug-loading capacity of these biomaterial systems enhances long-term therapeutic efficacy, improving the removal of residual tumor cells and reducing the risk of recurrence [46,47].

Hydrogels are another drug delivery method that has proven to have a lot of potential as an effective part of localized therapy. Their mechanical and physical characteristics can be finely adjusted by varying the polymer type, molecular weight, degree of crosslinking, or fabrication method. Meanwhile, their typical porous structure provides sufficient space for the effective entrapment of various therapeutic agents [48]. Hydrogels are typically maintained under physiological conditions but can be designed to respond to exogenous stimuli, e.g., temperature, light, or electric fields, as well as endogenous stimuli, e.g., acidic pH or redox conditions. This responsiveness enables the controlled, targeted release of encapsulated agents by structurally altering the hydrogel network. Furthermore, injectable biodegradable hydrogels that form a gel in situ have the advantage of fluid-like behavior, allowing them to adapt to tissue surfaces and fill irregularly shaped cavities, e.g., those left behind by breast tumor resection, thereby improving local delivery of therapeutic factors [49].

### 4.2. Types of Hydrogels

**Figure 4:**
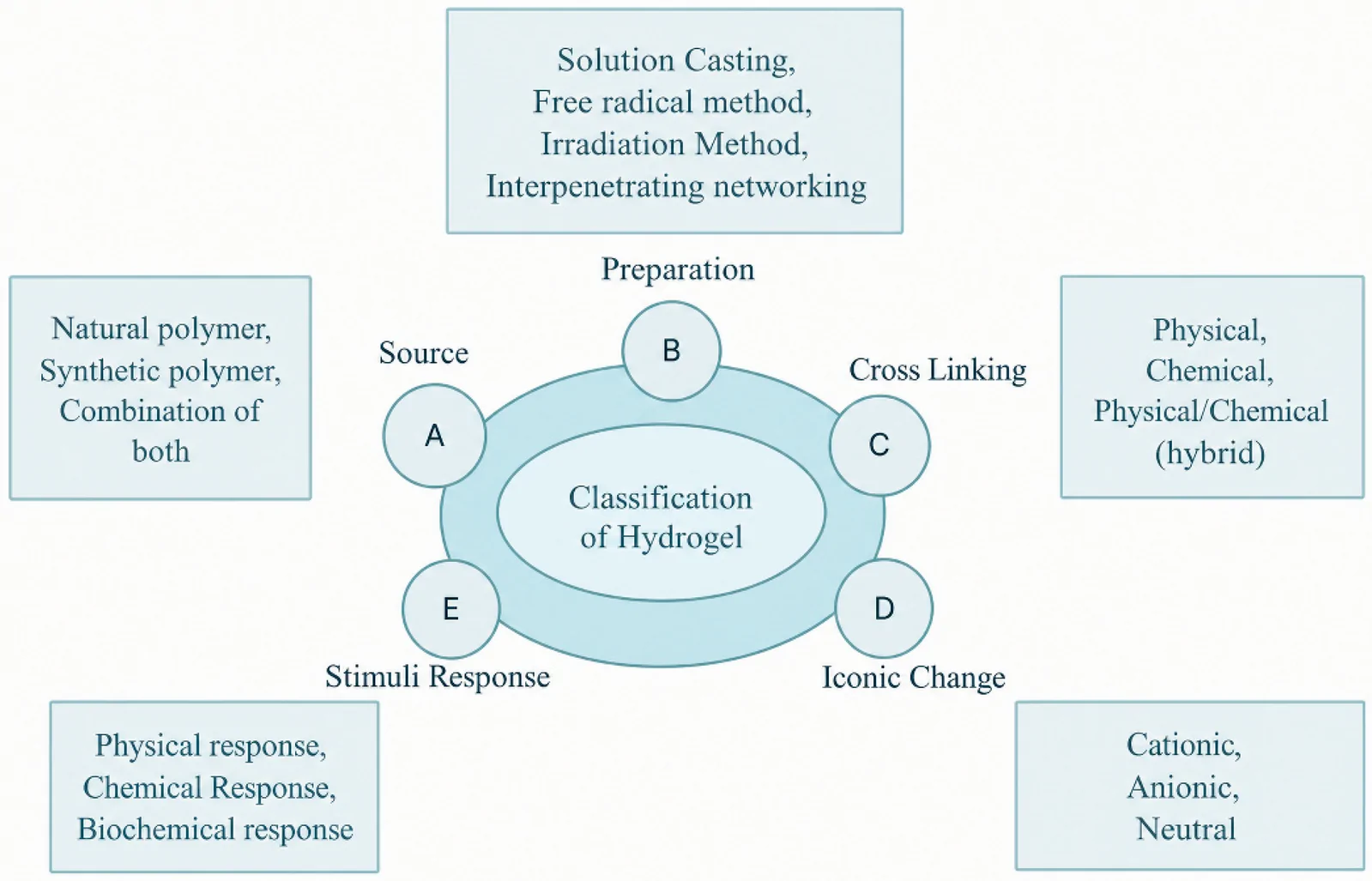
Classification of Hydrogel.

Hydrogels can be classified into three groups based on their polymeric roots: natural, synthetic, and hybrid [50,51] (Figure 4). The chemical composition and crosslinking of the hydrogel decide the mechanical strength, swelling, degradation, and drug release of the hydrogel. Consequently, natural, synthetic, or hybrid polymers should be chosen in view of the specific requirements of postoperative breast cancer therapy, such as adaptability to the resection cavity, sustained local delivery of drug, tissue acceptability, and controlled design. A careful comparison of these material qualities can help us understand how hydrogel composition can affect therapeutic efficacy and clinical translation [52,53].

**Figure 5:**
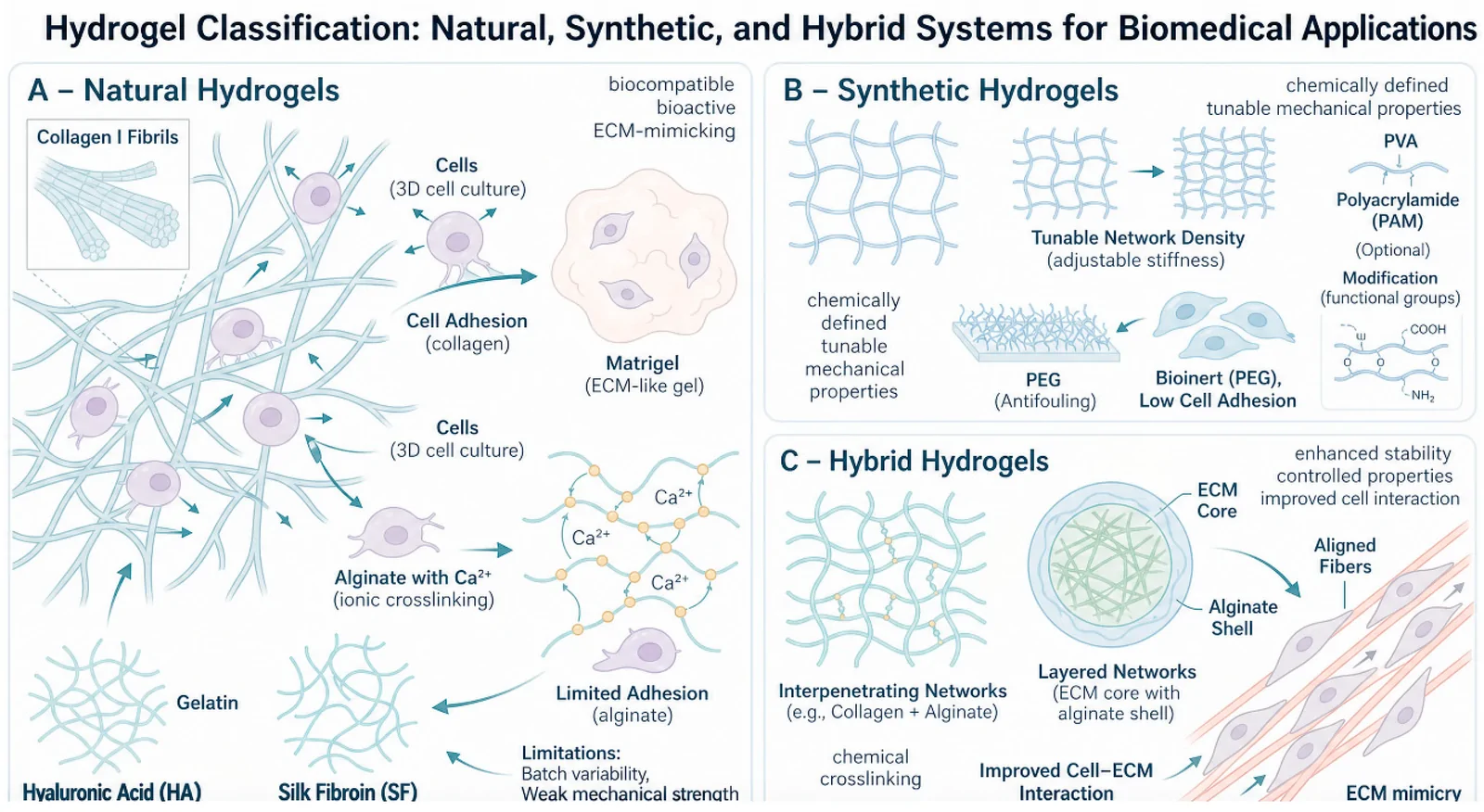
Advances in Hydrogel Design: From Natural to Hybrid Systems for Biomedical Applications.

In Figure 5. Advances in hydrogel design: from natural to hybrid systems for biomedical applications. (A) Natural hydrogels, including collagen, gelatin, hyaluronic acid (HA), silk fibroin (SF), and alginate. Collagen-based systems support cell adhesion and 3D cell culture, while alginate can undergo Ca^2+^-mediated ionic crosslinking but may show batch variability and limited mechanical strength. (B) Synthetic hydrogels, including polyethylene glycol (PEG), polyvinyl alcohol (PVA), and polyacrylamide (PAm), provide chemically defined networks with tunable network density and mechanical properties; PEG can provide antifouling and low cell-adhesion properties, while functional-group modification enables further customization. (C) Hybrid hydrogels combine natural and synthetic components through interpenetrating or layered networks, including ECM-core/alginate-shell structures and aligned fibers, to enhance stability, provide controlled properties, improve cell–ECM interactions, and mimic the native ECM.

#### 4.2.1. Natural Hydrogels

The extracellular matrix (ECM) of the body is particularly compatible with natural hydrogels due to their chemical composition and fibrous structure [54]. Natural hydrogels commonly used in the biomedical industry are Matrigel, collagen, alginate, silk fibrin (SF), hyaluronic acid (HA), and gelatin [55,56,57]. They find widespread application in biomedical research in 3D cell culture, microfluidic devices, biodegradable implants, and preclinical drug development [58].

Matrigel and collagen I have been regarded as the gold standards in 3D cell culture [59]. Matrigel is a common animal-derived extract of approximately 1500 different proteins, the most common of which are laminin, collagen IV, and entactin [60]. Matrigel is a gel that can form an ECM-like matrix at room temperature without sacrificing the biocompatibility and bioactivity needed to support cell growth and survival. Collagen I is a well-known extracellular matrix protein that can facilitate cell adhesion by binding to cell-surface receptors and other ECM proteins via its multiple binding sites [61,62]. Physical crosslinking is commonly used to form collagen hydrogels via pH- or temperature-mediated reactions, thereby creating three-dimensional fibrillary matrices that recapitulate the extracellular matrix milieu in vivo [63]. The composition and crosslinking mechanism of natural hydrogels strongly influence their mechanical stability, degradation rate, swelling behavior, and drug-release characteristics. For postoperative breast cancer applications, these properties are important for maintaining the hydrogel within the irregular resection cavity and providing sustained local delivery while supporting tissue compatibility. Natural-polymer composition and crosslinking regulate hydrogel stability, degradation, and drug release. In postoperative breast-cancer applications, these hydrogels may remain stable for approximately 2–4 weeks, with drug release lasting several weeks to 80 days. Increased crosslinking generally slows degradation and drug diffusion whereas reduced crosslinking accelerates both process [64].

Nevertheless, Matrigel and collagen are both highly disadvantageous due to their mechanical properties and batch-to-batch variability [65]. Notable natural hydrogels include alginate, gelatin, HA, and SF, each with unique properties. Alginate is a polysaccharide derived from brown algae, and its use in cell transplantation and wound healing is based on its low immunogenicity and high biocompatibility [66,67]. The simplest polymer that can readily entrap cells is alginate, a polymer composed of (1,4)-linked 2-D-mannuronic acid and alpha-L-guluronic acid blocks, which forms gels in Ca^2+^ solution. Calcium-alginate hydrogels exhibit poor cell adhesion because they lack binding sites, yet they exhibit high elasticity owing to an inter-grid structure formed by crosslinking ions [68].

#### 4.2.2. Synthetic Hydrogels

Synthetic hydrogels offer several advantages over natural hydrogels, including faster chemical modification and greater mechanical flexibility. Previous studies indicate that polyethylene glycol (PEG), polyvinyl alcohol (PVA), polyacrylamide (PAm), and polylactic acid (PLA) are common synthetic polymers in the biomedical sector [69]. PEG and its analogs are biocompatible and versatile; thus, PEG-based hydrogels can be used as typical synthetic hydrogels [70]. However, PEG is a hydrophilic, bioinert polymer that forms an antifouling coating, preventing adhesion receptors (integrins) from recognizing the cell surface. PVA is nontoxic, completely biodegradable, and a polymer best suited to form a hydrogel, as it has excellent film-forming properties and biocompatibility [71]. Nevertheless, PVA hydrogels have serious limitations, such as low heat retention and poor mechanical properties (Table 2). Cellulose nanofibrils and nanocellulose were used to improve the mechanical and thermal properties of PVA without reducing its biodegradability and transparency [72].

#### 4.2.3. Hybrid Hydrogels

Both natural and synthetic hydrogels are widely used, but a single type cannot meet all the needs of biomedical applications (Figure 5). Consequently, scientists have developed new hybrid hydrogels that combine multiple advantages into a single system to enhance matrix stability, reduce batch-to-batch variability, regulate responsiveness, and better mimic the original ECM. Indicatively, a collagen I11 collagen I3011 collagen I-Matrigel composite hydrogel was developed, and discovered that local fiber orientation increased cell-ECM interactions. A different example is the construction of composite living fibers (CLFs), which are a variant of functional microfibers with a central core of ECM proteins and a shell of Ca^2+^-alginate, produced by using textile technology to construct tissues [73].

The CLF allowed cell growth to occur in the optimal hydrogel layer surrounding the core region, and it had mechanical strength equal to that of a monofilament/multifilament core thread, biodegradable/nonbiodegradable suture thread. The internal strain field determined the formation of oriented collagen fibers in the composite hydrogels during the sandwiched ECM injection and Matrigel squeezing process, which forms a cell infiltration pattern similar to that observed in clinical biopsies of metastatic breast cancer [74].

Hybrid hydrogels combine natural and synthetic polymers to integrate biological functionality with improved mechanical and structural stability. The composition and ratio of the constituent polymers, together with their crosslinking interactions, can be adjusted to control stiffness, degradation, swelling, and therapeutic release. Such compositional flexibility may be particularly advantageous for postoperative breast cancer therapy, where both tissue compatibility and sustained local drug delivery are required. The extracellular matrix of most types of cancer, such as colon, breast, and prostate cancer, is stiffer than that of normal tissues [75,76]. Increased cell deformability or reduced stiffness has been associated with increased metastatic potential and invasiveness in cancer [77]. In a study of ovarian cancer cells of different invasiveness, the more invasive cell line was found to be more deformable than the less invasive cell line. Hydrogels are used to model the three-dimensional microenvironment of tissues to understand cell–cell interactions and intracellular signaling pathways (including proliferation, apoptosis, growth, and survival). The breast cancer tumor microenvironment exhibits altered ECM composition and mechanical properties, including increased stiffness. Hydrogels can mimic or modulate these properties through controlled polymer composition and network stiffness. These features are particularly relevant for studying tumor–matrix interactions and localized therapy after breast tumor resection. Recent studies have shown that the malignant nature of cancer cells is driven by mechanical signals arising from changes in their microenvironment. These mechanobiological cues that influence cancer invasion and proliferation are pressure, shear stress, and stiffness. All these mechanobiological tissue properties can be recapitulated by altering the hydrogels. The term stiffness is used interchangeably with elasticity and compliance [77]. The rigidity of hydrogels can generally be varied widely depending on the chemistry and base material. Hydrogels made of natural polymers tend to be lower in bulk stiffness (max elastic modulus of a few hundred Pa), and synthetic polymers have a greater range of stiffness of 10 s–100 s kPa [78,79]. Cells not only react to stiffness but also to the viscoelasticity of a material, as reflected in stress relaxation and nonlinear elasticity, including strain stiffening [80]. Recent studies have increasingly focused on hydrogel systems specifically designed for postoperative breast cancer therapy. These systems combine natural or synthetic polymer networks with tunable mechanical properties, bioadhesion, and controlled degradation to maintain the hydrogel within the resection cavity and provide sustained local delivery. Recent reviews have highlighted the use of advanced hydrogel platforms for breast cancer treatment, including drug-loaded, immunomodulatory, and tissue-engineering systems [10,81].

### 4.3. Synthesis and Crosslinking Strategies

Select hydrogel synthesis based on the intended application. Physically crosslinked systems offer rapid, reversible gelation, whereas covalently crosslinked systems provide greater stability. Injectable hydrogels typically gel within seconds to minutes, have moduli of approximately 1–10 kPa, and support drug release over weeks to months, depending on their composition and crosslinking density [64]. For postoperative breast cancer therapy, crosslinking should be designed to provide adequate mechanical stability, injectability, tissue retention, controlled degradation, and sustained drug release within the irregular resection cavity. Crosslinking density and network architecture directly influence these properties and therefore affect therapeutic performance [10,29].

#### 4.3.1. Physical Crosslinking

Physical crosslinking is frequently employed and relies on reversible non-covalent interactions, such as hydrogen bonding, ionic attractions, and hydrophobic interactions, which enable hydrogels to swell or shrink in response to environmental stimuli [82]. Representative physically crosslinked hydrogels include poly(vinyl alcohol) (PVA) formed through freeze–thaw-induced crystallization, alginate crosslinked ionically with Ca^2+^ ions, and chitosan-based hydrogels formed through electrostatic interactions with anionic polymers or molecules. These systems demonstrate how polymer chemistry and intermolecular interactions can be used to form stable hydrogel networks without covalent crosslinkers [83,84]. These physical networks can arise from hydrogen bonding, hydrophobic interactions, electrostatic interactions, host–guest interactions, or metal–ligand coordination. Such reversible interactions can provide shear-thinning and self-healing properties, which are particularly useful for injectable hydrogels that must conform to irregular postoperative cavities [29].

#### 4.3.2. Chemical Crosslinking

Chemical crosslinking entails the formation of permanent covalent bonds between polymer chains. Similar mechanisms include bulk polymerization, in which monomers are polymerized in the presence of free radicals generated by initiators to form mechanically stable hydrogel networks [85].

#### 4.3.3. Irradiation-Based Crosslinking

Also, irradiation-based methods, including ultraviolet (UV) exposure and electron beam (e) irradiation, are used to facilitate covalent crosslinking of the polymer matrix, thus enhancing structural stability and durability [85]. However, irradiation, chemical crosslinkers, and reaction conditions can also cause cytotoxicity and compromise the biocompatibility of these hydrogels [86].

## 5. Advanced Functional Hydrogels for Cancer Immunotherapy

### 5.1. Hybrid Hydrogels

Hybrid hydrogels specifically use a blend of physical and chemical crosslinking, with reversible interactions (e.g., hydrogen bonding) alongside irreversible covalent bonds. The two-network design offers greater mechanical stability and environmental responsiveness, which broadens its use in immunotherapeutic applications [87]. An example is a hybrid hydrogel that was reported by Phan et al., which consists of a thermosensitive poly(ε -caprolactone -co -lactide)-b-poly(ethylene glycol)-b-poly(ε -caprolactone -co -lactide) (PCLA) triblock copolymer mixed with bovine serum albumin (BSA). This system formed a hydrogel around the dermal area and served as a depot that localized the delivery of DNA to dendritic cells (DCs). The hydrated and porous protein-polymer network facilitated rapid infiltration of DCs without additional recruitment factors and enabled sustained DNA release. Significantly, this hydrogel preparation can be delivered via a minimally invasive injection using a standard hypodermic needle [88]. A recent study further demonstrated the potential of an injectable alginate-based hybrid hydrogel for localized HER2-positive breast cancer therapy, in which pH- and NIR-responsive mechanisms enabled controlled therapeutic release and combined chemotherapy, antibody therapy, and photothermal treatment.

After injection, the aqueous PCLA–BSA formulation undergoes thermally induced sol–gel transition at physiological temperature (~37 °C), driven primarily by increased hydrophobic interactions and self-assembly of the amphiphilic PCLA blocks. This transition converts the injectable polymer solution into a stable three-dimensional hydrogel network at the surgical site, enabling localized and sustained drug retention [89].

Another effective method to enhance the performance of a hydrogel in cancer immunotherapy is the incorporation of functional host molecules, including cyclodextrins. Supramolecular interactions involving cyclodextrin facilitate drug loading and controlled release of cargo. Devi et al. developed a hybrid hydrogel composed of thiol-modified hyaluronic acid and vinyl sulfone-functionalized 2-cyclodextrin. These hydrogels exhibited tumor-specific degradation and release profiles that could be tuned, making them ideal for site-specific immunotherapeutic delivery [90]. In postoperative tumor sites, degradation specificity can be achieved by incorporating bonds or linkages that respond to tumor-associated conditions, such as acidic pH or elevated ROS levels. Such stimulus-responsive degradation can accelerate polymer-network breakdown and drug release preferentially within the tumor microenvironment while maintaining greater stability under normal physiological conditions [18,29].

### 5.2. Stimuli-Responsive (Smart) Hydrogels

Crosslinking mechanisms can be engineered to respond to external or internal stimuli, such as temperature, pH, reactive oxygen species (ROS), or electric fields, to enhance hydrogel functionality. Smart hydrogels are stimuli-responsive, exhibiting dynamic changes in swelling behavior, degradation rate, or drug-release kinetics in response to environmental stimuli. This versatility renders them especially appealing for controlling in situ gel formation and achieving fine spatiotemporal control of therapeutic release in cancer immunotherapy [85].

### 5.3. Targeted Hydrogel Systems

Another important design consideration is targeted modulation of immune cells (Table 3). Hydrogels may be functionalized with ligands or molecules that bind to dendritic cell, lymph node, or cytotoxic T lymphocyte surface receptors, such as Toll-like receptors (TLRs), tumor necrosis factor receptor family members, C-type lectin receptors, and integrins. For example, thermosensitive mPEG–PLGA hydrogels containing granulocyte-macrophage colony-stimulating factor (GM-CSF) have been shown to stimulate the recruitment, proliferation, and maturation of DCs and macrophages locally. Also, hydrogels have the potential to serve as carriers for lymph-node-targeted nanoparticles [10]. Nanoparticles with the right physicochemical characteristics, including a neutral or slightly negative surface charge and a 10–200 nm diameter, can be efficiently drained and retained in lymph nodes, enhancing immune responses [91].

### 5.4. Drug Delivery and Its Release Kinetics Timeline

**Figure 6:**
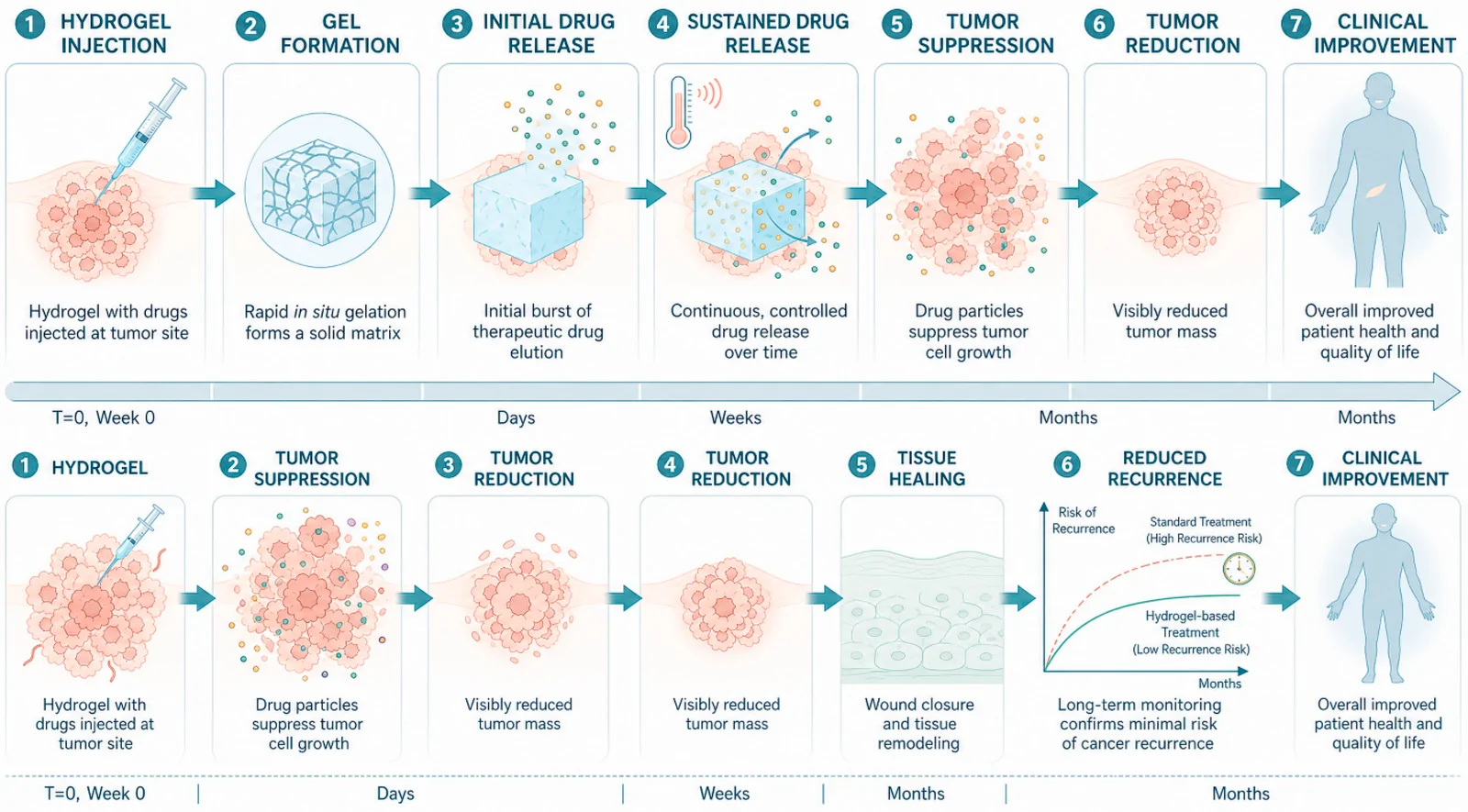
Hydrogel drug delivery for tumor treatment: A Timeline of clinical treatment.

Hydrogels have attracted a lot of attention due to their unique physical properties for use in drug delivery [93]. The porosity of hydrogels can be easily adjusted by controlling their water affinity and the density of crosslinkages. Also, medicines can be packed and then discharged due to porosity [94]. One of the advantages of hydrogels in drug delivery applications is the potential for sustained release of an active pharmaceutical ingredient, maintaining a high local concentration for a long period. Once the medication is embedded in a hydrogel, it may be released via diffusion-controlled, swelling-controlled, chemically controlled, or environmentally responsive mechanisms [95]. The drug release kinetics timeline is also revealed in this review paper (Figure 6).

### 5.5. Hydrogel Delivery Approaches and Clinical Challenges

The clinical translation of hydrogel-based therapies depends not only on their therapeutic effects but also on effective delivery and stable depot formation at the target site. Their injectability and biocompatibility are important advantages; however, the route of administration and associated barriers are also critical factors. For breast cancer, intratumoral and intraoperative administration allow a flowable hydrogel precursor to be delivered directly into the tumor or post-resection cavity, followed by in situ gelation through physiological cues such as temperature, pH, ionic interactions, or chemical crosslinking. This transition from a flowable precursor to a three-dimensional network enables the hydrogel to conform to irregular tissue geometry and provide localized drug retention. Properties such as shear-thinning, rapid gelation, tissue adhesion, and self-healing can further improve injectability and structural stability. Intraoperative application may also allow hydrogel precursors to be sprayed, injected, or implanted into the resection bed immediately after excision, providing direct coverage of irregular surgical surfaces [96].

These advantages are limited by the localized hydrogel depots. The major disadvantages of intratumoral or intraoperative delivery are its invasive nature and reliance on anatomical constraints [93,97]. Not eligible are disseminated metastatic disease, hematological malignancies, and deeply located, incurable solid tumors whose direct approach is dangerous or not possible.

In Figure 6, describe the temporal progression of hydrogel-based drug delivery and its potential therapeutic outcomes. The upper part illustrates the drug-delivery process following hydrogel injection, including rapid in situ gel formation, initial drug release, sustained drug release, and subsequent tumor suppression and reduction. This sequence highlights the role of the hydrogel as a localized drug reservoir that enables prolonged therapeutic exposure at the target site [95]. In contrast, the lower part emphasizes the longer-term biological and clinical consequences of localized hydrogel therapy, progressing from tumor suppression and tumor reduction to tissue healing, reduced recurrence risk, and overall clinical improvement. Thus, the upper and lower sections distinguish the drug-delivery and pharmacological sequence from the subsequent therapeutic, tissue-repair, and clinical outcomes, respectively [47,96].

Moreover, the changing and diverse nature of the TME could compromise the performance of a fixed depot. A static depot could result in early drug degradation, burst rupture, or an uneven distribution of drugs due to variable enzyme activity levels, pH gradients, and elevated interstitial fluid pressure within the TME [52,98]. Another challenge is achieving reliable, steady performance in vivo. The complex in vivo environment can influence drug release kinetics and depot lifespan and alter the gelation kinetics, ultimate mechanical strength, and degradation profile observed in vitro. The hydrogel carrier, either because it is too sticky or disintegrates too early, may lead to therapeutic failure [99]. For postoperative applications, thermosensitive polymers such as PLGA–PEG–PLGA can remain flowable at room temperature and undergo sol–gel transition near physiological temperature, enabling minimally invasive injection and conformal filling of irregular resection cavities. However, increasing polymer concentration or crosslinking can improve mechanical stability and drug retention at the expense of injectability and diffusion [10,29].

## 6. Discussion and Future Perspectives

The continuing problem of tumor recurrence after surgery, especially in breast cancer, is a constant issue and is the main reason why more effective and less toxic treatment methods are urgently required. Although surgical resection is the mainstay of therapy, microscopic residual disease and the counterintuitive pro-tumorigenic impact of surgical stress frequently restrict its capacity to eradicate malignant cells [14,16,17]. The traditional systemic adjuvant treatments, despite their common use, are limited by nonselective biodistribution and severe off-target toxicities [6,7,24].

**Figure 7:**
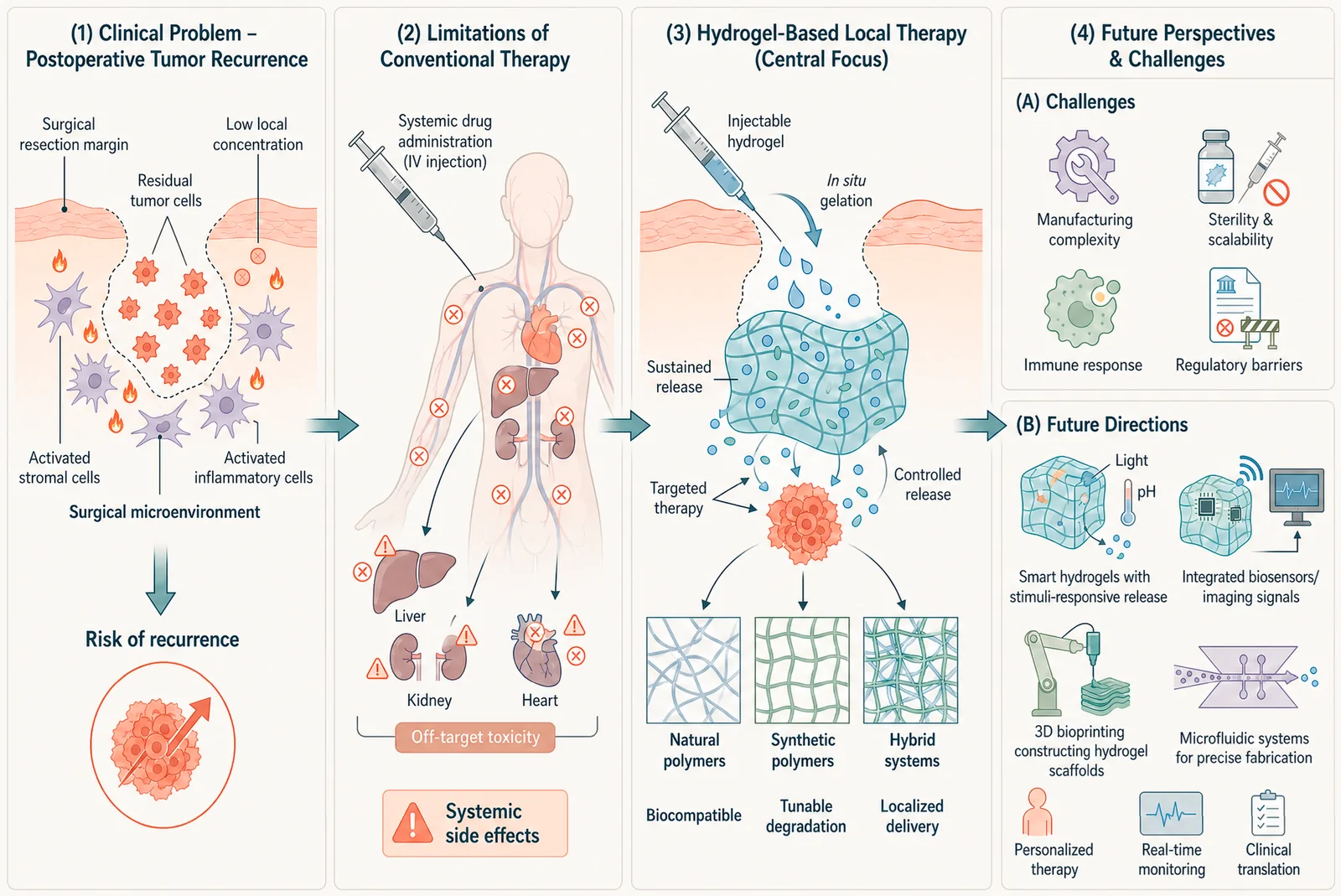
Summary of Hydrogel-Based Local Drug Delivery Systems for Preventing Postoperative Breast Cancer Recurrence.

Local drug delivery systems based on hydrogels are an interesting alternative. Their distinct features, such as biocompatibility, tunable mechanical and degradation behaviors, and the ability to in situ gel, make them particularly suitable for filling irregular surgical cavities and delivering sustained, localized anticancer agents [45,47] The potential to prepare hydrogels using natural, synthetic, or hybrid polymers enables a high degree of customization to fit the needs of clinical application, with highly nuanced strategies of functionalization enabling stimuli-responsive and targeted delivery, which further increases therapeutic effectiveness [85,87,88]. This customization is achieved by varying polymer composition, molecular weight, crosslinking density, and network architecture to regulate mechanical strength, degradation, swelling, drug loading, and release kinetics. Functionalization with stimuli-responsive groups can further enable hydrogel responses to tumor-associated pH, ROS, enzymes, or external temperature/light, thereby providing controlled and localized therapeutic release [29,100]. Although promising developments have been made, several challenges remain in translating hydrogel-based therapies into common clinical practice. The challenge of producing reproducible, scalable, and sterile hydrogel formulations needs to be addressed. Long-term safety profiles, such as the potential for chronic inflammation or foreign body reactions, need to be thoroughly investigated through well-designed preclinical and clinical trials [86]. Also, regulatory routes for combination products (hydrogel + drug) are developing and could be a challenge to commercialization [29] (Figure 7). From a polymer-material perspective, these challenges are closely related to controlling polymer composition, molecular weight, crosslinking density, degradation behavior, and batch-to-batch consistency, which can directly affect gelation, mechanical properties, drug release, and in vivo safety. Therefore, standardized fabrication and characterization are essential for reproducible hydrogel performance and successful clinical translation [10,101].

Figure 7 provides an integrated overview of the clinical rationale, therapeutic strategy, and translational considerations associated with hydrogel-based local drug delivery for preventing postoperative breast cancer recurrence. Part (1) summarizes the clinical problem of postoperative tumor recurrence, including residual tumor cells and surgery-induced changes in the local microenvironment that may contribute to tumor regrowth and metastasis. Part (2) highlights the limitations of conventional systemic adjuvant therapies, including nonspecific biodistribution, limited drug accumulation at the surgical site, and systemic toxicity. Part (3) illustrates hydrogel-based localized therapy, in which injectable and in situ-forming hydrogels can conform to irregular resection cavities and provide sustained and controlled release of therapeutic agents. The use of natural, synthetic, and hybrid polymeric networks further enables modulation of hydrogel properties, degradation, mechanical characteristics, and therapeutic functionality. Part (4) summarizes the major challenges and future opportunities for clinical translation. Panel (A) highlights challenges related to hydrogel fabrication and reproducibility, sterilization, scalability, biocompatibility, immune responses, and regulatory requirements, whereas panel (B) presents future directions including stimuli-responsive systems, integrated imaging or biosensing, 3D bioprinting, microfluidic fabrication, personalized therapy, and real-time monitoring.

Future studies ought to emphasize the creation of multifunctional hydrogels that can incorporate therapeutic delivery and real-time monitoring capabilities, such as imaging or biosensing, to enable personalized management of postoperative care [14,66]. The precision and reproducibility of hydrogel constructs may be further increased through the incorporation of emerging technologies such as 3D bioprinting and microfluidics [11,55]. In addition, clinical trials on the safety and efficacy of hydrogel-based local therapies on breast cancer patients will be necessary to determine their place in the standard-of-care paradigm [102].

## 7. Conclusions

Tumor recurrence following surgery is a daunting problem in cancer treatment, especially in breast cancer, where lingering malignant cells and postsurgical pro-tumorigenic stress induced by the surgery play a role in disease recurrence. The traditional systemic adjuvant therapies have constraints of low bioavailability, off-target toxicity, and insufficient drug concentration at the tumor bed. An alternative, yet equally enticing, approach is the use of hydrogel-based local drug delivery systems, which enable sustained, controlled, and localized release of therapeutic agents at the surgical site. The range of hydrogel materials, based on natural, synthetic, or hybrid polymers, enables the specific design of their physicochemical characteristics, degradation rate, and sensitivity to environmental factors. Further applications in cancer immunotherapy and personalized postoperative care have been realized through advanced functionalization schemes, such as hybrid network design, stimuli-responsive behavior, and immune-cell targeting. However, the mechanistic evidence discussed in this review is primarily derived from individual hydrogel systems and their specific therapeutic applications rather than from a unified mechanistic framework. Therefore, further systematic studies are needed to clarify the molecular and material mechanisms linking hydrogel composition, crosslinking, drug release, and therapeutic outcomes in postoperative breast cancer. In situ-forming and injectable hydrogels are of particular interest for filling irregular resection cavities and for minimally invasive delivery. Although there is positive preclinical evidence, scalability, reproducibility, long-term safety, and regulatory approval remain issues that need to be addressed before translation into clinical practice. Investigations should be directed toward multimodal systems that incorporate therapeutic delivery and real-time monitoring, as well as carefully designed clinical trials that verify efficacy in patients. Through future interdisciplinary cooperation, hydrogel-based systems have significant potential to transform postoperative care, reduce cancer recurrence, and ultimately improve the quality of life of cancer survivors. Overall, localized hydrogel therapeutics show strong potential for preventing postoperative breast cancer recurrence through sustained local drug delivery. Tunable composition, crosslinking, and stimuli-responsive release can improve treatment at the resection site. Further breast cancer-specific studies are needed to support clinical translation.

## Figures and Tables

**Table 1:** Limitations of Primary Adjuvant and Surgical Modalities in Breast Cancer.

Modality	Core Limitations in Breast Cancer	Common Adverse Effects	References
Chemotherapy	Nonspecific toxicity; low tumor targeting (<1%); induction of multidrug resistance (MDR).	Myelosuppression, cardiotoxicity, nausea, and alopecia.	[31]
Radiotherapy	Limited to the radiation field; risk of damage to adjacent organs (heart/lungs).	Radiation dermatitis, localized fibrosis, and lymphedema.	[32]
Immunotherapy	Low response rates in hormone-positive subtypes; high cost of treatment.	Immune-related adverse events (irAEs) such as thyroiditis, colitis, and pneumonitis.	[33]
Surgery	Potential for Minimal Residual Disease (MRD); surgical stress may trigger tumor regrowth.	Surgical site infections (SSIs), seromas, and aesthetic defects/tissue voids.	[26]

**Table 2:** Comparative characteristics of natural, synthetic, and hybrid hydrogels for postoperative breast cancer applications.

Hydrogel Type	Representative Polymers	Key Properties	Main Limitations	Relevance to Postoperative Breast Cancer
Natural	Collagen, alginate, HA, gelatin	Biocompatibility, ECM-like environment, biodegradability	Weak mechanical stability, variable degradation	Tissue compatibility and local drug delivery
Synthetic	PEG, PVA, PAm	Tunable mechanical properties, controlled degradation and drug release	Limited intrinsic bioactivity	Sustained drug delivery and structural stability
Hybrid	Natural + synthetic polymers	Combines biological functionality with mechanical stability	More complex fabrication and characterization	Improved balance between tissue compatibility and sustained local therapy

**Table 3:** Hydrogel Synthesis and Functional Strategies for Cancer Immunotherapy.

Aspect	Type/Strategy	Key Features	Relevance to Cancer Immunotherapy	Refs
Hydrogel Synthesis	Physical crosslinking	Physical crosslinking through repeated freeze–thaw cycles; reversible hydrogen-bonding interactions between PVA chains; pH- and NIR-responsive swelling and drug release.	Provides a stable local drug depot for sustained chemotherapy and photothermal therapy in postoperative breast cancer.	[9]
	Chemical crosslinking	Covalent crosslinking through Schiff-base reactions between functionalized polymer chains; forms stable three-dimensional networks with controlled degradation and sustained drug release.	Provides an injectable and stable local drug-delivery platform for sustained chemotherapy in breast cancer.	[92]
	Irradiation-based crosslinking	UV- or e-beam-induced covalent bonding; enhanced durability	Improves mechanical strength but may induce cytotoxicity	[82,85,86]
Functional Enhancement	Hybrid hydrogels	Combination of physical + chemical crosslinking; improved stability and responsiveness	Versatile platforms for controlled immune cargo delivery	[87]
	Protein–polymer hybrid hydrogels	Thermosensitive PCLA–BSA system; in situ gel formation; microporous network	Enables dendritic cell infiltration and sustained DNA delivery	[88]
	Cyclodextrin-based hybrid hydrogels	β-cyclodextrin inclusion complexes; supramolecular drug encapsulation	Enhances loading capacity and controlled, tumor-responsive release	[90]
Stimuli-Responsive	Smart hydrogels	Respond to pH, temperature, ROS, electric field, and dynamic swelling/degradation.	Allow on-demand drug release and in situ modulation	[85]
Targeted Systems	DC-targeted hydrogels	Incorporation of ligands for TLRs, TNF receptors, lectins, and integrins	Enhances DC recruitment, activation, and immune priming	[91]
	Lymph node–targeted systems	Integration of nanoparticles (10–200 nm, neutral/negative charge)	Promotes lymphatic drainage and efficient immune activation	
Administration	Injectable hydrogels	In situ gelation via hypodermic needle; minimally invasive	Improves patient compliance and localized immune modulation.	

## Data Availability

No new data were created or analyzed in this study. Data sharing is not applicable to this article.
